# Rats did not show evidence of prospective information-seeking: a pilot study

**DOI:** 10.3389/fnbeh.2023.1253780

**Published:** 2023-12-04

**Authors:** Sumie Iwasaki, Tohru Taniuchi

**Affiliations:** Institute of Human and Social Sciences, Kanazawa University, Kanazawa, Japan

**Keywords:** information-seeking, rats, metacognition, prospective behavior, exploratory behavior

## Abstract

Information-seeking behavior often features in research on metacognition in non-human animals; some species seek more information when they do not know the location of a food reward. Rats are known to do this in situations of uncertainty, but it is still unclear if they seek information prospectively for solving a later problem. In this study, we investigated rats’ information-seeking responses in two areas that presented different cognitive challenges (*N* = 4). In one area, a memory task was presented in which rats could access a cue for a food reward during the information-seeking phase of a trial, but the cue was removed before the subsequent test phase. In the other area, a discrimination task presented a cue that was available in both the information-seeking and the test phases, so that it was not necessary to seek information prospectively. The memory and discrimination test trials were given in quasi-random order (Experiment 1). Rats explored in the memory task area no more than in the discrimination task area during the information-seeking phase, even after extensive training. When they were exposed exclusively to the memory task over multiple sessions (Experiment 2), they developed a strategy of exploring the available object cues. In Experiment 3, rats were found to stay longer in an area, which had an object than in other, less potentially informative areas; they were sensitive to the presence of information. Although these results did not support the existence of prospective information-seeking in rats, they do not necessarily imply that rats lack related abilities. This consideration is due to the constraints of the small sample size and the limited scope of the testing environment. Accumulating not only positive but also negative evidence would further understanding of the factors influencing metacognitive responses in non-human animals.

## 1 Introduction

Information-seeking to solve a task features prominently in research on metacognition in non-human animals. Because information-seeking or checking behavior in foraging contexts is both naturalistic and useful for finding food (Call, 2012), metacognition paradigms using this behavior have been applied in studies of various animal species (apes, Call and Carpenter, 2001; Suda-King, 2008; Call, 2010; Marsh and MacDonald, 2012, lion-tailed macaques, Marsh, 2014, rhesus monkeys, Hampton et al., 2004, capuchin monkeys, Paukner et al., 2006; Basile et al., 2009; Vining and Marsh, 2015, lemurs, Taylor et al., 2020, rats, Roberts et al., 2012; Kirk et al., 2014, ravens, Lambert and Osvath, 2020, scrub-jays, Watanabe et al., 2014; Watanabe and Clayton, 2016, and canines, Bräuer et al., 2004; McMahon et al., 2010; Belger and Brauer, 2018; Royka et al., 2020). Some species have shown information-seeking depending on their knowledge states, in the form of checking or looking at potential food reward-containing locations when ignorant of the food’s whereabouts, but accessing it immediately when aware of its location. Furthermore, comparative studies have revealed differences in information-seeking pattern among species, reward values, and experimental settings (e.g., Call, 2010; Beran and Smith, 2011; Iwasaki et al., 2013, 2018).

Whereas numerous studies have investigated animals’ information-seeking behaviors while solving a task, few studies have focused on prospective information-seeking. Watanabe et al. (2014) showed that scrub-jays spent more time studying information before solving a difficult problem than an easy one. Subjects were required to find food rewards that were hidden in two compartments; one compartment had four potential hiding places (open cups), while the other had only one open cup. The subjects could look into the compartments through peepholes, while an experimenter simultaneously hid rewards in cups in both compartments. It was found that the birds looked more frequently and for longer into the compartment containing four open cups than the compartment with only one open cup. This finding indicates that scrub-jays allocated their study time depending on the task, suggesting prospective information-seeking. Similar results have been reported in behavioral studies of human children (Iwasaki et al., 2020; Brinums et al., 2021). Prospective information-seeking is related not only to the ability to monitor own current knowledge states, but also to future-oriented cognition. Accumulation of evidence for prospective information-seeking in various species could help reveal its phylogenetic distribution, but so far we have few data, from a small number of species.

Kirk et al. (2014) reported that rats sought information to solve a T-maze and a radial maze task. Rats were trained to press a lever at the central choice point to obtain a food pellet. Pressing the lever also provided an illuminating cue to the location of a second reward at the end of one of the alleys. After rats learned to press the lever and to obtain both rewards, as a test the first rewards were omitted. If rats pressed the lever only to obtain the first reward and not for information about the location of a second reward, lever pressing should be extinguished. However, even without the immediate reinforcement of the first reward, rats continued to press the lever. Moreover, in the eight-arm radial maze, lever pressing occurred at a higher rate than in the T-maze. These findings suggest an effect of task difficulty in determining the value of the cue in these levers pressing tasks, and that rats actively seek information about location of rewards.

Studies have reported that rats made prospective judgments according to strength or certainty of their memory (Foote and Crystal, 2007; Templer et al., 2017; Yuki and Okanoya, 2017). In a modified matching to sample task in which rats were allowed to choose between taking a matching test involving comparison stimuli or avoiding it, rats tended to prefer the latter option when their memory accuracy was low due to a long delay or omission of presentation of the sample. By contrast, rats tended to take the matching test when their memory accuracy was high. These studies suggested that rats could monitor their current memory states and control their behavior before responding.

**Figure 1:**
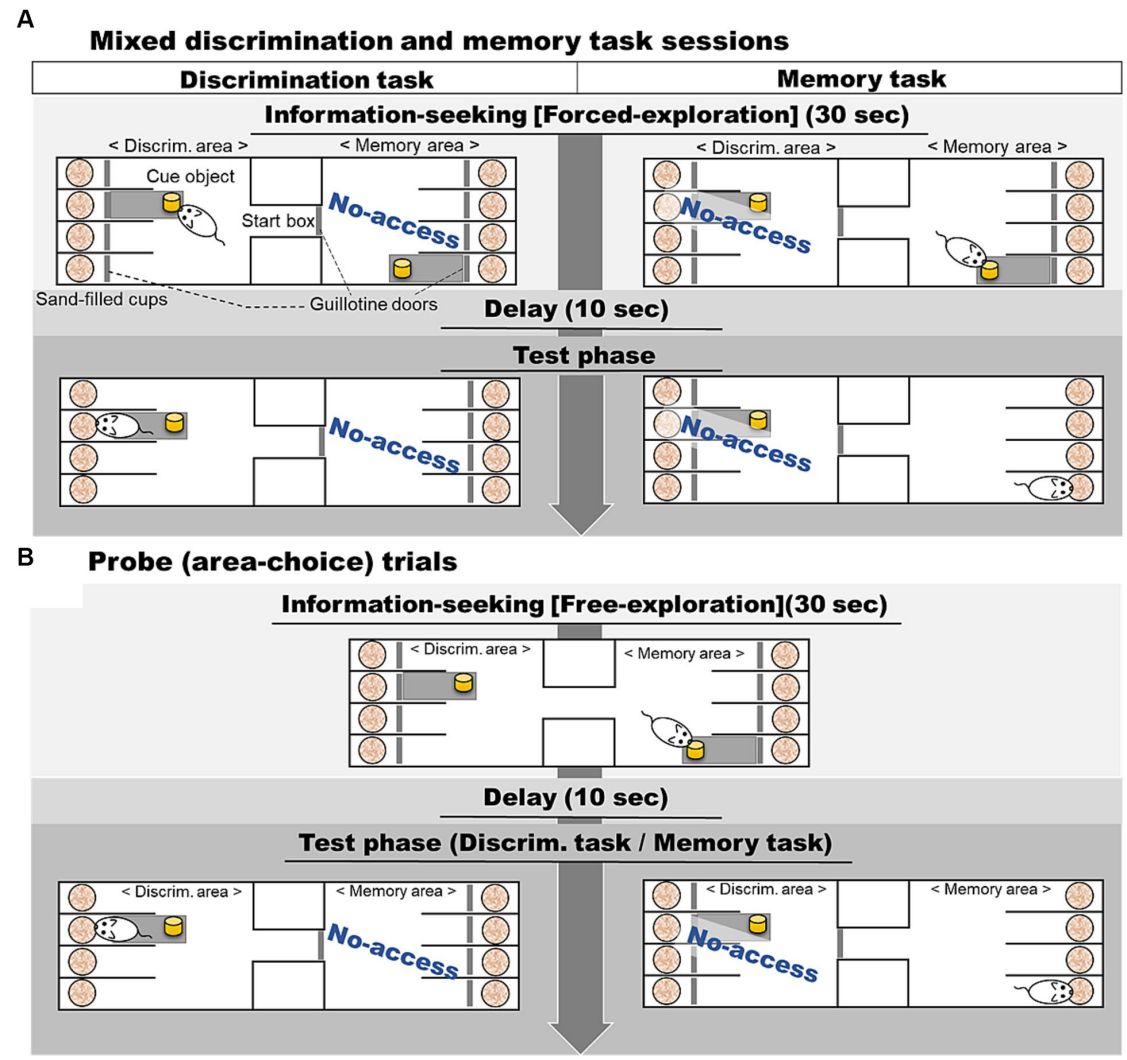
A flow diagram illustrating **(A)** the mixed discrimination and memory task sessions and **(B)** the probe trials. In the mixed discrimination and memory task sessions, rats were allowed to explore a designated area (the memory area or discrimination area) for a duration of 30-s in the information-seeking phase. In the probe trials, rats were allowed to freely explore both side areas during the information-seeking phase. After a 10-s delay, rats were presented with either a discrimination task, where a cue was presented, or a memory task, where no cue was available. A trial was considered complete when rats dug in one of the four sand-filled cups and either consumed a food reward (in the case of correct trials) or began digging in one of the three incorrect cups (in the case of incorrect trials).

In this study, we investigated rats’ prospective information-seeking responses in two areas (Figure 1), based on an experimental design used in studies of birds and human children (Watanabe et al., 2014; Iwasaki et al., 2020). In the memory area, rats could access a cue for a food reward during an initial information-seeking phase, but the cue was removed before the test phase, which involved a memory task. In the discrimination area, a cue was available in the information-seeking phase and the discrimination test phase, eliminating the need to seek information during the first phase. The two areas were fixed throughout the study. During training subjects were forced to explore only one area in each trial, in an arbitrary order. In probe trials they were allowed to explore the both areas during the information-seeking phase, that is, they could choose to seek information in neither area, in one area, or in both areas. We analyzed the rats’ exploring behavior (duration of sniffing of cue objects) as an information-seeking behavior in probe trials. If rats sought information prospectively for the later test phase, they should explore the cue object for longer in the memory area than in the discrimination area during the information-seeking phase.

## 2 Experiment 1a: tests of prospective information-seeking

In Experiment1a, we investigated rats’ prospective information-seeking responses in two situations: in the discrimination test area, a cue for solving the task was available in both the pre-test and the test phase, but in the memory test area, the cue was removed in advance of the test phase.

### 2.1 Methods

#### 2.1.1 Subjects

The subjects were four naïve male Sprague–Dawley (SD) rats, purchased from Charles River Laboratories Japan (Yokohama, Japan) when they were approximately 70 days old. They were not littermates. Each rat was housed individually in a plastic cage measuring 276 mm × 445 mm × 204 mm. The breeding room maintained a 12:12 reversed light: dark cycle, with the lights turning on at 8:00 a.m. For the 30 days after the introduction, both food (from Funabashi Farm, Chiba, Japan; MM-3) and water were available *ad-libitum* in their home cages. From the start to the end of the experiment, rats were restricted to a daily ration of 16 g of food to ensure their weight remained consistently at least 95% of their *ad-libitum* weight. The rats were transported from their home cages to the experimental room in a wire-mesh cage divided into five compartments, each measuring 150 mm × 210 mm × 170 mm. This cage also served as a waiting area during the experiments, which were conducted between 10:00 a.m. and 1:00 p.m., 5 days a week. After each day’s experiment, a 16 g food ration was provided in their home cages, at least 15 min post-experiment. Throughout the study, water was available *ad-libitum* in their home cages. The care and use of the rats adhered to the guidelines provided by Kanazawa University, and the experimental procedures were approved by Kanazawa University Animal Experimentation Regulations.

#### 2.1.2 Apparatus

The apparatus, made of gray PVC (120 cm long, 40.9 cm wide, and 40 cm high) was divided into three separate areas (see Figure 1). The center area was a start box (19.4 cm × 10 cm), from which subjects could enter the two side areas when the guillotine doors were open. At each end of the apparatus, four cups (7.5 cm diameter, 8 cm tall) filled with sand were sunk into the floor and separated from each other by 20 cm-high walls. A food reward (250 mg chocolate flavored cereal, Kellogg, Chiyoda, Japan, chokowa) was hidden at the bottom of one of these cups. PVC doors (9.5 cm wide, 30 cm high) in front of the cups could prevent subjects from accessing them.

Cues that could be used to locate rewards consisted of two sets of eight objects differing in color, shape, and material (e.g., plastic or porcelain; see Supplementary Figure 1). Each object was fixed to a PVC base (9.8 cm wide, 19.3 cm long). Cues were presented only once per session to prevent proactive interference across daily trials.

#### 2.1.3 Procedures

##### 2.1.3.1 Pretraining

For the first 20 days of the pretraining, each rat was handled for 1 min every day. Starting on Day 21, they began their habituation to the apparatus. During the first 4 days of the habituation, individual rats were allowed to explore the apparatus for 15 min each day. For this familiarization, both guillotine doors were open, allowing access to the entire apparatus. A chocolate-flavored cereal was placed on the surface of all sand-filled cups and distributed in both side areas. After familiarization, rats were trained to dig in the sand to obtain a food reward. During this pretraining phase, only one PVC door in front of the cups was open; the others remained closed. First, a reward was placed on the surface of the sand. An experimenter put the rat into the start box, and then opened both guillotine doors. The rat entered one area and obtained the reward from the accessible cup. After the rat obtained the reward reliably, the reward was buried progressively deeper in the sand. In this way, all rats were trained to obtain rewards from the bottom of the cups. One session was conducted each day, and each session consisted of eight trials, once per reward cup, presented in pseudo-random order. This training was completed within 14–18 days.

##### 2.1.3.2 Discrimination task

In the discrimination task, rats were required to choose only the baited and therefore correct cup, which was directly behind a cue-object. The cue-object was placed directly in front of the correct cup, and no other object was in front of any of the other (incorrect) cups. The task always took place in the same side area (the “discrimination area”), which was counterbalanced across subjects. After a guillotine door in start box was opened, rats were immediately required to choose one of four cups. During first four sessions, rats were prompted to dig in the correct cup because the doors to the three remaining cups were closed. From session 5, all doors were open, meaning that rats could respond to any of the four cups. A chocolate-flavored cereal was placed in all cups to mask any potential odor cues. The cup in which the subject dug first was deemed as the choice. Rats were allowed to dig in the correct cup to obtain the reward, but they were removed from the apparatus immediately if they started digging in an incorrect cup, preventing them from getting the food. Incorrect trials were followed by up to two correction trials, which were repetitions of the first trial. If a second correction trial was necessary, plastic covers were placed over the incorrect cups to prevent digging, thereby channeling the rat to the correct cup. Daily sessions consisted of eight trials, one trial per object, presented in random order. The position of the correct cup was allocated pseudo-randomly; the same correct position did not occur in two consecutive trials. Rats were trained until they performed at above 80% correct (chance level was 25%) in two consecutive sessions blocks. All rats reached criterion within 6–12 sessions.

##### 2.1.3.3 Memory task

The memory task consisted of three phases: exploring a sample (cue) object, a short delay, and then a matching-to-place test without the object. The door to the “memory area,” which was on the opposite side of the start box to the discrimination area, was opened so that the rat could explore the object. The location of the object marked the correct cup, but during this information-seeking phase the closed door in front of the cup prevented access to it. After the 30-s the information-seeking phase, the rat was returned to the start box and remained there during the 10-s delay, during which the experimenter removed the object and opened the doors in front of all four cups. After the delay, the door of the start box was opened and the rat was allowed to re-enter the memory area to choose a cup. As the object was no longer present in the test phase, any choice of the previously cued cup might indicate that the rat remembered the cue location during the information-seeking phase. Daily sessions consisted of eight trials, with all other aspects of procedure identical to those for the discrimination task. The rats were trained until they scored above 60% correct (chance level was 25%) in two consecutive sessions. All rats reached criterion within 6–14 sessions.

##### 2.1.3.4 Mixed discrimination and memory task sessions

Mixed sessions consisted of four discrimination and memory trials each, presented in pseudo-random order to avoid presenting the same task in three consecutive trials. In the sessions, regardless of task type, rats were always allowed to explore the appropriate area with a sample object for 30-s (information-seeking phase) and then waited in the start box for 10-s (delay phase; Figure 1A). In the discrimination task, the cue object was left in position, available in the test phase, whereas in the memory task, the cue object was removed before the test phase. Allocation of areas for the discrimination and memory tasks was as for training. We ran a total of 8 mixed sessions (total: 32 trials per task).

##### 2.1.3.5 Probe (area choice) trials

In probe trials, procedures were almost the same as in mixed session training, except that rats had a choice of which area to explore during the information-seeking phase. In the trials (Figure 1B), the two doors of the start box opened simultaneously, allowing rats to explore both side areas freely for the next 30-s. The same cue objects were placed in both areas. During the information-seeking phase in probe trials, rats had no knowledge of which task would appear in the subsequent test phase. Each session included six training trials and two probe trials; the latter were inserted randomly except that the occurrence of two probe trials in succession was avoided. We ran eight sessions, yielding test data from 16 probe trials for each subject.

#### 2.1.4 Data analysis

All statistical analyses were performed using R (version 4.1.2). Statistical tests were considered significant if *p* < 0.05.

In coding of rats’ behavior, we defined the start of the information-seeking phase as the moment that both guillotine doors adjacent to the areas left the floor. We coded approaching to within 2 cm of the object as exploratory behavior; approaches were often/usually but not always accompanied by sniffing. QuickTime Player (Apple Inc.) was used for this coding. A second coder, unaware of the purpose of the study, coded 25% of all trials. We calculated a concordance rate using the Pearson correlation coefficient and the correlation between the durations of exploration coded by one of the authors and by the second coder was very high (*r* = 0.98).

In the training trials, to compare exploration times toward objects in the memory and discrimination areas, we used a two-tailed paired *t*-test. For each probe trial, we computed a difference score by subtracting the exploration duration of objects in the discrimination area from that in the memory area. A positive score indicated a longer exploration time in the memory area, consistent with prospective information-seeking. To compare the group mean with a chance level of 0, we estimated a 95% confidence interval (CI) based on the differences in exploration durations of four subjects. Finally, to assess whether exploration during the information-seeking phase improved the rats’ performance in the tasks, we compared the percentage of correct responses with or without exploration to a chance level of 25% using a two-tailed binomial test.

### 2.2 Results and discussion

**Figure 2:**
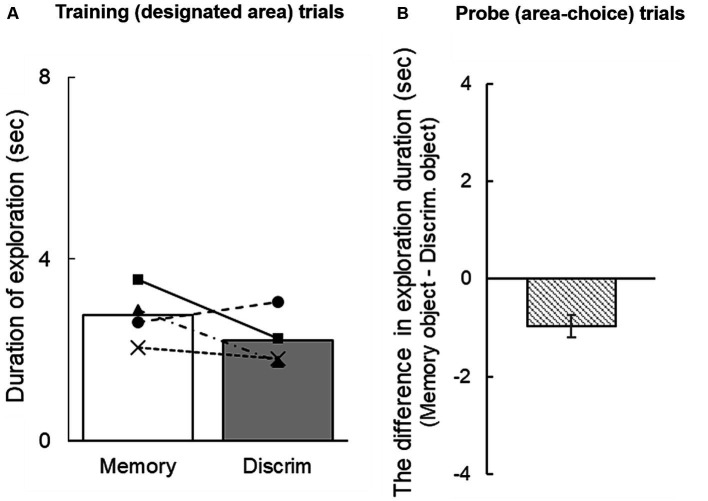
**(A)** Duration of exploration of a sample object during the information-seeking phase in training trials. **(B)** Mean difference in exploration duration in probe trials. A negative difference score indicates longer exploration of the object in the discrimination area than in the memory area. The error bar indicates 95% CI estimated from data of four subjects.

Figure 2A shows the duration of exploring each cue during the information-seeking phase in training trials. Exploration time toward the objects did not differ significantly between the memory and discrimination areas [*t*(3) = 1.36, n.s.]. In the information-seeking phase of the probe trials, individual subjects’ mean difference in exploration durations between the memory and discrimination areas were − 0.82, −1.12, −1.02, and − 0.86. The group mean was −0.96 [95% CI: −1.18 to −0.73] (Figure 2B): rats explored the objects for significantly longer in the discrimination area than in the memory area.

Probe trials in which subjects did not approach an object in the memory area during the information-seeking phase (nine trials in total) yielded an accuracy score of 22.22%, very close to the 25% chance level. By contrast, when they did explore objects in the memory area (23 trials in total), memory accuracy was 56.52%, significantly above chance (*p* < 0.05). Discrimination accuracy in test trials was 100%, regardless of object exploration in the discrimination area (exploration occurred in 21 trials, and was absent in in 11 trials). These results suggested that exploring the cue object during the information-seeking phase enhanced rats’ performance in the memory task, but not the discrimination task.

In Experiment 1a, we investigated rats’ prospective information-seeking responses to two areas. If rats sought information prospectively for solving a task, they would explore a cue longer in the memory area than in the discrimination area in probe trials. Contrary to our prediction, rats explored cue objects more in the discrimination area, where information-seeking was unnecessary for the subsequent test. The subjects’ preference for this area might be explained by the greater success in the discrimination task than that in the memory task (100 vs. 46.87% correct, respectively). Thus, in Experiment 1a, we found no evidence of prospective information-seeking in rats. Adams and Santi (2011) reported that extensive training eventually resulted in pigeons responding adaptively in relation to their memory states, whereas such responses were not observed during initial testing. Would more extensive training enhance effective information-seeking behavior in rats? In Experiment 1b, we repeated area-choice trials which were the same as probe trials in Experiment 1a, and then examined whether rats showed prospective information-seeking behavior.

## 3 Experiment 1b: effects of extended training on prospective information-seeking

In Experiment 1b, we investigated the effects of extended training on prospective information-seeking by repeating the area-choice trials (i.e., probe trials) as in Experiment 1a.

### 3.1 Methods

#### 3.1.1 Subjects and apparatus

Subjects and an apparatus were same as in Experiment 1a.

#### 3.1.2 Procedures

Procedures and cording were almost the same as in Experiment 1a, except that sessions consisted entirely of all area-choice trials. One day after finishing Experiment 1a, we began extended training of the rats for Experiment 1b, conducting eight sessions (total trials: 64).

#### 3.1.3 Data analysis

The correlation in duration coding between one author and a second coder was *r* = 0.91, with this reliability test covering 25% of the total trials. A one-way repeated measures ANOVA was applied to analyze the transitions in the difference in exploration duration toward the cues in the two areas. Other aspects of the data coding and statistical analysis were identical to those of Experiment 1a.

### 3.2 Results and discussion

**Figure 3:**
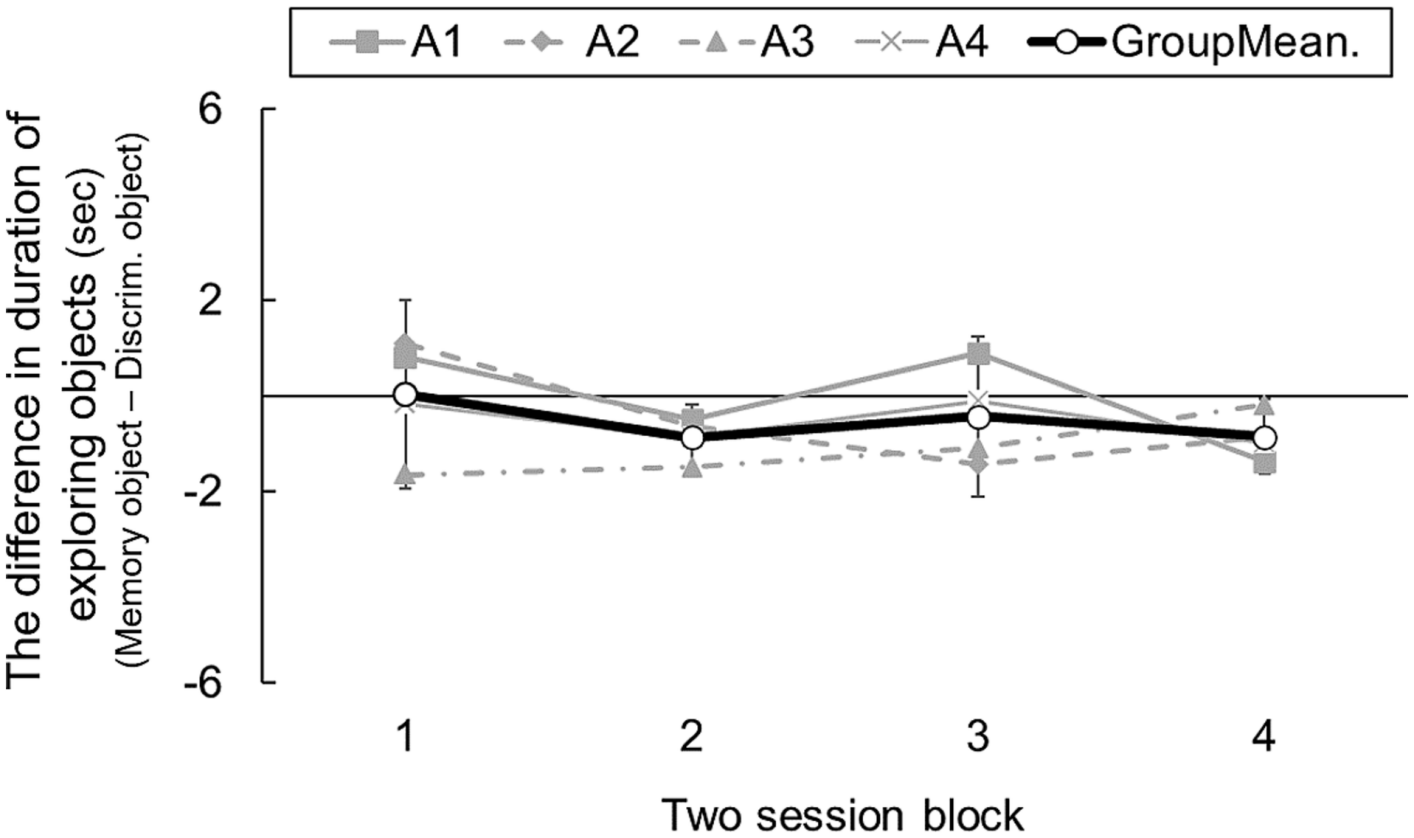
The difference in duration of exploring cue objects in two-session blocks, which consisted of open area-choice trials (Experiment 1b). Error bars indicate 95% CI estimated from data of four subjects.

Figure 3 shows transitions in the difference in exploration duration toward the cue objects cues in the two areas. A one-way ANOVA revealed a nonsignificant main effect of blocks [*F*(3, 9) = 0.90, n.s.], indicating that rats developed no tendency to seek information for longer in the memory area than in the discrimination area. Moreover, individual mean duration differences were − 0.02, −0.45, −1.09, and − 0.52. The group mean was −0.51 [95% CI: −1.21 – 0.17]. Based on this result, rats did not explore the object cues for longer in the memory area than in the discrimination area. Although 64 area-choice trials were run, rats showed no evidence of prospective information-seeking.

Area-choice trials in which subjects did not approach an object in the memory area during the information-seeking phase (14 trials in total) yielded an accuracy score of 35.71%, which was not statistically significant compared to the chance level of 25%. When they did explore objects in the memory area during the information-seeking phase (114 trials in total), memory accuracy was 61.40% (*p* < 0.05, two-tailed binomial test). In contrast, the accuracy score was significantly higher than the chance level of 25% not only when exploration toward the object in the discrimination area occurred during the information-seeking phase (97.29%, 111 trials in total) but also when it was absent (100%, 17 trials in total).

Although in Experiment 1a, the rats showed a significant preference for exploring discrimination area objects, no such preference was seen in the first 2-session block of Experiment 1b: rats explored objects equally in the two areas [95% CI: −1.94 – 2.00]. The reason for the different results in Experiments 1a and 1b is unclear, but repeated probe trials might have resulted in rats recognizing the difference in conditions, and developing a slight tendency to seek information in the memory area. However, the most salient finding so far was that rats did not seek information for longer in the memory area than in the discrimination area, a finding that contrasts with what has been reported in human children and birds (Watanabe et al., 2014; Iwasaki et al., 2020). While subjects in previous studies were given opportunities to solve both tasks after their information-seeking, our subjects were assigned to solve either one task or the other. Brinums et al. (2021) informed children which sets of cards would be brought back later and found that they studied or touched those sets they knew would be used in later tests. Knowing which tasks will be performed later may enhance relevant information-seeking.

In Experiment 2, memory tasks were presented exclusively, giving rats the opportunity to learn about the occurrence of the memory task in the future test phase of a trial. During the information-seeking phase rats were allowed to explore the objects in the both areas.

## 4 Experiment 2: information-seeking when only memory tasks are presented

In Experiment 2, we examined whether rats sought information prospectively for the later test phase when this involved memory tasks only.

### 4.1 Methods

#### 4.1.1 Subjects and apparatus

Subjects and an apparatus were same as in Experiment 1a.

#### 4.1.2 Procedures

Procedures and coding were almost that same as in Experiment 1b, except that only the memory task was conducted in all trials. Rats were allowed to explore in both areas in the information-seeking phase, but only presented with the memory task in the test phase. We conducted 12 sessions (total trials: 96).

#### 4.1.3 Data analysis

The correlation in duration coding between one author and a second coder was *r* = 0.95, with this reliability test covering 25% of the total trials. The methods of data coding and statistical analyses were identical to those used in Experiments 1a and 1b.

### 4.2 Results and discussion

Trials in which subjects did not approach an object in the memory area during the information-seeking phase (28 trials in total) yielded an accuracy score of 32.14%, which was not statistically significant compared to the chance level of 25%. When they did explore objects in the memory area during the information-seeking phase (356 trials in total), memory accuracy was 57.30% (*p* < 0.05).

**Figure 4:**
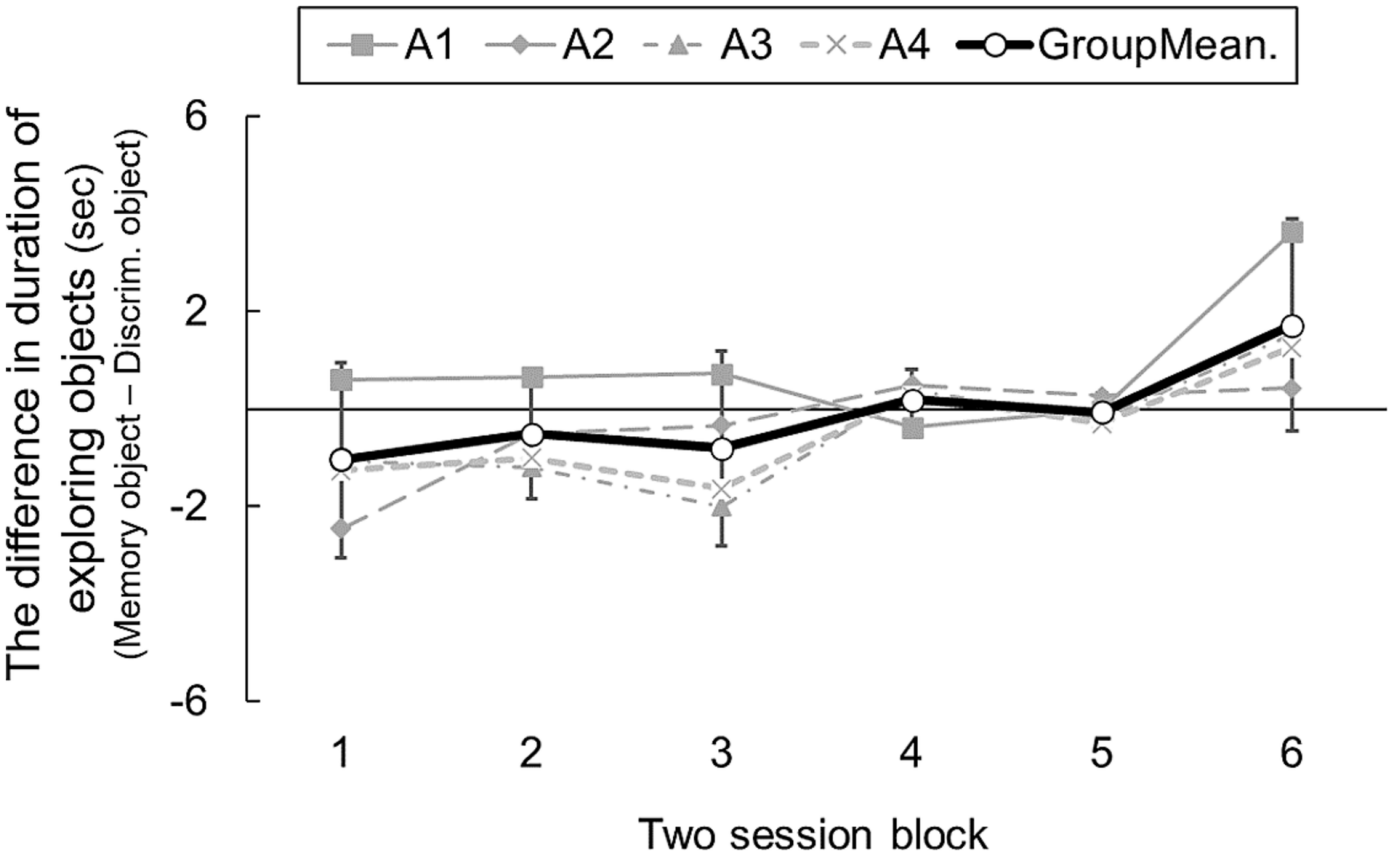
The difference duration of exploring cue objects in sessions consisting of all choice trials and presenting only the memory task (Experiment 2). Error bars indicate 95% CI estimated from data of four subjects.

Figure 4 show transitions in differences of cue object exploration in two-session blocks. A one-way ANOVA revealed a significant main effect of blocks [*F*(5, 15) = 5.73, *p* = 0.003]. *Post hoc* analysis indicated that rats explored the memory area for longer in the final, sixth block than the first block [*t*(3) = 22.49, *p* = 0.002, adjusted by Shaffer’s method]. This result shows that the subjects learned to seek information in the memory area over sessions. However, even in the final block, the group mean was 1.71 [95% CI: −0.45 – 3.88]: rats did not explore the object in the memory area significantly above chance. Experiment 2 might therefore have revealed weak evidence for prospective information-seeking in rats, but conceivably, exploring the memory area during the information-seeking phase was reinforced by the food reward in the test phase in the multiple area-choice trials (e.g., Smith et al., 2009). In that case, rats might not recognize which information was needed for solving later tasks.

Although the rats tended to explore the memory area object more over sessions, they did not do so at more than chance level, even by the final block. In this task, knowing which task would be presented later appeared to enhance appropriate information-seeking in rats, albeit to a limited extent. In a final experiment, we examined whether rats would engage in information-seeking differentially according to the presence or absence of information. During the information-seeking phase, an object was placed in the memory area only, never in the discrimination area (no information area). We analyzed the duration of remaining in each area. If rats respond to information, they should stay longer in the memory area, containing a cue object, than in the discrimination area, with no such information.

## 5 Experiment 3: information-seeking according to the presence of information

In Experiment 3, we examined whether rats would respond to availability of information. We predicted that they would stay longer in an area containing a cue object than in an area which had no cue-related information.

### 5.1 Methods

#### 5.1.1 Subjects and apparatus

Subjects and an apparatus were the same as in Experiment 2.

#### 5.1.2 Procedures

Procedures were almost same as in Experiment 2, except that a cue object was placed only in the memory area and not in the discrimination area during the information-seeking phase. We conducted 12 sessions (total trials: 96).

#### 5.1.3 Data analysis

In Experiment 3, we could not compare exploration behavior based on sniffing duration against the object stimuli, as there was no object in the discrimination area. Therefore, the analysis focused on the length of time spent in each area. A rat was considered present in an area as long as the tip of its nose was within that area. The correlation in duration coding between one author and a second coder was *r* = 0.98, with this reliability test covering 25% of the total trials. Other aspects of the statistical analysis were identical to those in Experiments 1a, 1b, and 2.

### 5.2 Results and discussion

**Figure 5:**
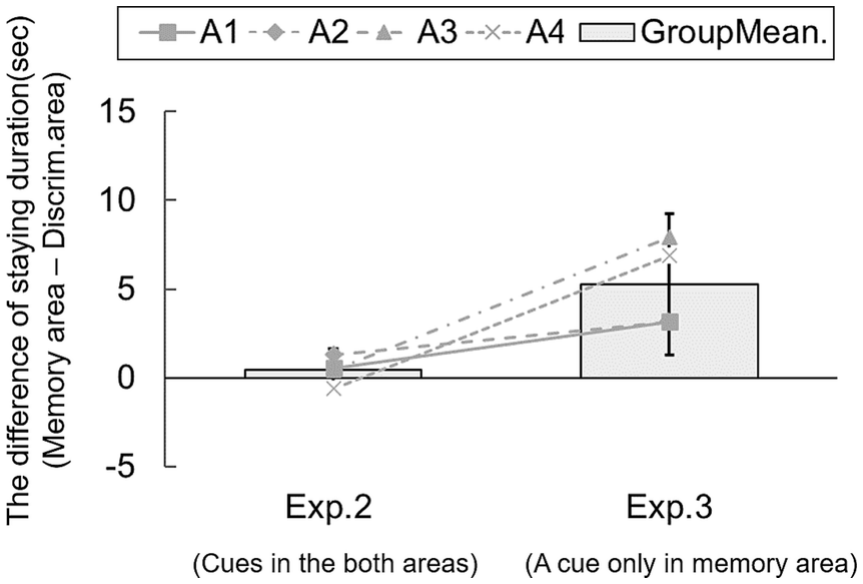
Comparison of the time spent in memory and discrimination areas in Experiments 2 and 3. Lines show individual data. Error bars indicate 95% CI estimated from data of four subjects.

Across all trials (384 trials in total), subjects explored in the memory area (cue-object area) during the information phase, and the accuracy score was 73.43% (*p* < 0.05). We calculated the difference in duration of time spent in the areas for each trial. Figure 5 shows the group means of the difference between memory and discrimination areas in Experiments 2 and 3. Although the difference score in Experiment 2 did not differ from chance [95% CI: −0.82 – 1.66], the corresponding score in Experiment 3 was significantly above chance [95% CI: 1.30–9.23]. A one-way ANOVA revealed no significant main effect of block [*F*(5, 15) = 0.16, n.s.], suggesting no development of exploration in the area over blocks (see Supplementary Figure 2B). Results showed that rats stayed longer in the area containing future-relevant information than in an area with no such information, suggesting sensitivity to the current availability of information.

## 6 General discussion

We examined whether rats sought information prospectively, for solving a task in the future. Before presentation of the task, they were allowed to explore and seek information in two areas: a discrimination area in which a cue to the location of food would be available in both the information-seeking and test phases, and a memory area in which the cue was available only in the information-seeking phase, being removed before the test phase. If rats showed relevant information-seeking, they should explore cues more in the memory area. Results did not support this prediction, as rats engaged in more information seeking in the discrimination area than in the memory area in occasional probe test trials (Experiment 1a). Even with 64 area-choice trials (Experiment 1b), rats continued to explore the two areas almost equally. When only the memory task was presented (Experiment 2), rats tended to increase their exploration of the cue object in the memory area over sessions. However, this learning effect could be explained by association between information-seeking behavior and food rewards during repeated area-choice trials (Smith et al., 2009), challenging any claim for prospective information-seeking in rats in these tests. Moreover, in Experiment 2, we found no exploration above chance even in the final training block. In summary, rats did not demonstrate a preference for exploring a necessary cue that would be useful for them in solving a later problem.

In contrast with our findings are reports of prospective information-seeking in human children and scrub-jays, which allocated more to studying time for an upcoming hard task than an easy task (Watanabe et al., 2014; Iwasaki et al., 2020). However, it seems too early to conclude that the literature supports species differences in prospective information-seeking. In this study, information-seeking behavior was similar to maze arm- or alley-selecting responses with regard to approaching or entering them. We interpreted approaching an object during the information-seeking phase as an information-seeking response, but perhaps rats simply attempted to choose the cup behind the cue object, or to solve the task during this phase. The similarity between the information-seeking and goal-choice behaviors might have reduced the likelihood of clearer positive results in this study. In a previous study by Watanabe et al. (2014), which demonstrated positive evidence of prospective information-seeking in scrub jays, subjects were prompted to peek through a hole in a partition, characterizing it as an information-seeking behavior. In contrast, they were only allowed to approach a reward cup during the goal-choice phase. The introduction of a partition with a hole during the information-seeking phase effectively distinguished information-seeking behaviors from goal-choice behaviors. Future studies might consider distinguishing between cue-directed information-seeking behaviors, like peeping through a hole in a partition, and the exhibited choice behavior when presented with goal cups.

In this study, rats did not seek information prospectively, but they did respond to the presence of information. Experiment 3 showed that rats explored an area with information relevant to the task for longer than another area without such information. Rats are reported to respond to obtain information about the location of food (Kirk et al., 2014). Our results suggest that rats are sensitive to the availability of potentially relevant information.

In this study, we used exploratory behavior as a sign of information-seeking responses, as it is one of the standard behavioral indicators in research on rodents (e.g., Winters et al., 2008; Fonio et al., 2009; Binder et al., 2012; Betsuyaku et al., 2016; Thompson et al., 2018); furthermore, it requires little in the way of training. Numerous studies have shown that exploratory duration and frequency may increase in unpredictable or uncertain situations. Moreover, exploration of potentially informative stimuli may be related to curiosity and task-related information-seeking (van Lieshout et al., 2020). From this viewpoint, it is reasonable to suggest that that information-seeking studies in rodents should include more investigation of their object-directed exploratory behavior. However, exploratory behavior may also be driven by a negative affective state, with the aim of reducing uncertainty (van Lieshout et al., 2020; Verjat et al., 2021). Such a negative motivation might apply to our results, which showed almost equal exploration in the memory and discrimination areas. However, the present study does not allow us to determine which type of motivation drove the exploratory behaviors. Future research on information-seeking in animals might therefore aim to reveal not only metacognitive abilities, but also reveal more about their motivations for seeking information.

A limitation is that our study used only four subjects. If we tested a larger number of rats in this task, a few rats might have exhibited potential prospective information-seeking responses. Therefore, increasing the number of subjects is essential to reach a definitive conclusion regarding the rats’ abilities. However, given the consistent response tendencies observed in all four rats, our results strongly indicate that the majority of rats may not display adaptive information-seeking behavior in our experimental setting, even if they have the ability to do so. Primal studies of metacognition in pigeons used three subjects and failed to show their metacognitive responses (Inman and Shettleworth, 1999; Sole et al., 2003; Sutton and Shettleworth, 2008), but the later studies inspired by these studies demonstrated positive evidence by using the different task or situations (Nakamura et al., 2011; Castro and Wasserman, 2013; Iwasaki et al., 2013, 2018). Our preliminary findings could also contribute to the refinement of experimental settings, as well as stimulate further research, discussions, and constructive criticism. In this study, we used Sprague Dawley rats due to their documented metacognitive responses in a pioneering study by Foote and Crystal (2007). However, research has also provided evidence of metacognition in Long Evans rats (Templer et al., 2017; Yuki and Okanoya, 2017; Joo et al., 2021). Moreover, sex differences in rat metacognition studies remain unexplored, as previous research have investigated only male subjects. Therefore, it is important to examine the generality of our findings across different rat strains (e.g., Long Evans rats) and rats of different sexes.

It should be noted that the successful information-seeking behavior in this experiment setup can be interpreted in various ways without the future oriented cognition. Especially, the prolonged examination of the sample object in the memory task area has the potential to enhance working memory, leading to an increased frequency of reinforcements. Consequently, these reinforcements could contribute to the heightened exploratory behavior observed in the memory task area. To address this, we need to introduce additional control conditions, such as controlling for reinforcement history. For example, in the information-seeking group, similar to our current study, exploratory behavior directed at an object cue can provide the necessary information for a subsequent discrimination task. In contrast, in the control group, rats will not engage in a discrimination task. Instead, their exploratory behaviors toward an object (which does not serve as a cue for the following task) during the initial phase will be reinforced by the experimenter in a later phase. Any increase in exploratory behavior in the control group can be attributed to the effect of positive reinforcement. If the frequency of exploratory behavior in the information-seeking group surpasses that in the control group, it could be interpreted as genuine information-seeking behavior. Iwasaki et al. (2020), employing a similar paradigm, proposed an alternative interpretation suggesting that subjects behaved in accordance with their current knowledge or curiosity, rather than anticipating their future knowledge state or an upcoming event. In order to refute this possibility, evidence is needed to show that present knowledge may not guarantee the successful resolution of a future task.

In this pilot study, we found no evidence of prospective information-seeking in rats, assessed by their exploration of cue objects relevant to a later test. They explored two areas equally regardless of the value of information-seeking for the later test, which contrasts with results of studies on scrub-jays and human-children (Watanabe et al., 2014; Iwasaki et al., 2020). Many studies have focused on information-seeking to examine metacognition in non-human animals because it is a naturalistic response in foraging contexts. Moreover, it has been suggested that prospective information-seeking might be related to future-oriented cognition (Iwasaki and Kishimoto, 2021). Accumulation of not only positive but also negative evidence concerning prospective information-seeking in diverse species can help to construct a true picture of the phylogenetic distribution of this cognitive ability across species.

## Data availability statement

The original contributions presented in the study are included in the article/Supplementary material, further inquiries can be directed to the corresponding author.

## Ethics statement

The animal study was approved by Committee on Animal Experimentation of Kanazawa University (AP-204194). The study was conducted in accordance with the local legislation and institutional requirements.

## Author contributions

SI: Writing – original draft, Writing – review & editing. TT: Writing – original draft, Writing – review & editing.
