# Supplementary material for: Rats did not show evidence of prospective information-seeking: a pilot study
Source: Front Behav Neurosci. 2023 Dec 4;17:1253780. doi: 10.3389/fnbeh.2023.1253780 (PMC10725935; doi:10.3389/fnbeh.2023.1253780)
Supplement: Supplementary file 1 [file Data_Sheet_1.docx]

Supplementary Material

Rats did not show preference for exploring the necessary cue for solving a later problem: Absence of prospective information-seeking?

Sumie Iwasaki*, Tohru Taniuchi

*** Correspondence:** Sumie Iwasaki: siwasaki623@staff.kanazawa-u.ac.jp

# Supplementary Figures


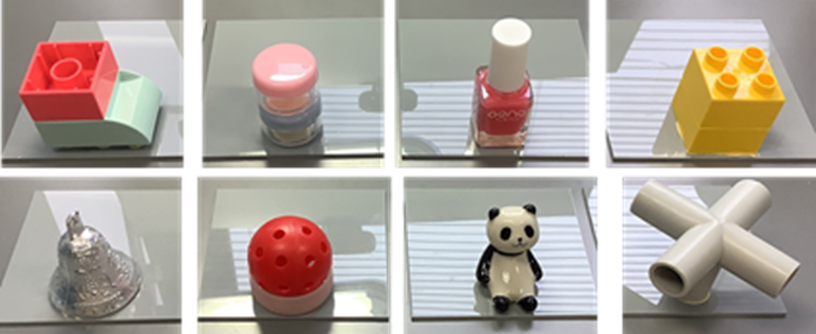


**Supplementary Figure 1.** Objects used as cues for locating food rewards.


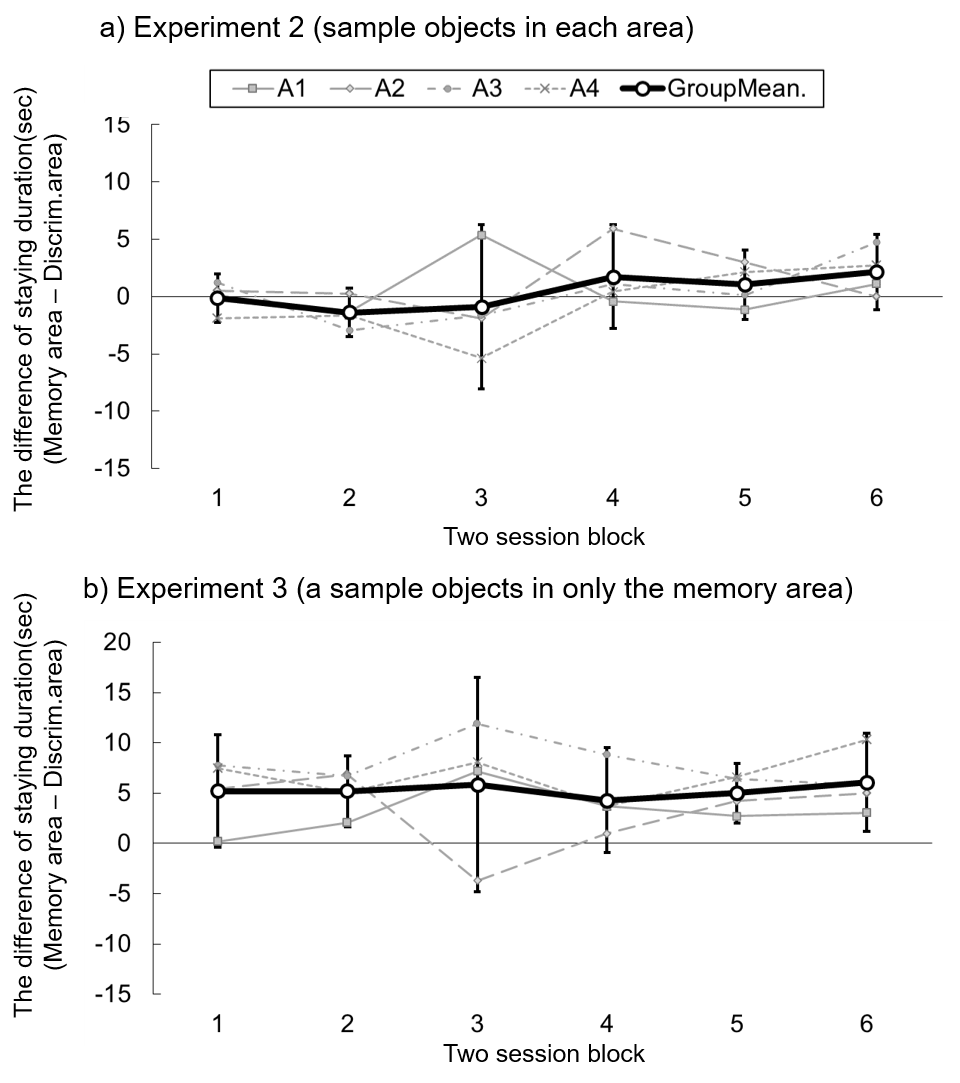


**Supplementary Figure 2.** The difference in duration of time spent in the two areas in Experiment 2 (a), where cue objects were present in both areas for the information-seeking phase, and in Experiment 3 (b), where a cue object was present in the memory area only.
